# Tumor matrix stiffness provides fertile soil for cancer stem cells

**DOI:** 10.1186/s12935-023-02992-w

**Published:** 2023-07-20

**Authors:** Sadegh Safaei, Roya Sajed, Ahmad Shariftabrizi, Shima Dorafshan, Leili Saeednejad Zanjani, Masoumeh Dehghan Manshadi, Zahra Madjd, Roya Ghods

**Affiliations:** 1 Department of Molecular Medicine, Faculty of Advanced Technologies in Medicine, Iran University of Medical Sciences, Hemmat Street (Highway), Next to Milad Tower, Tehran, 14496-14530 Iran; 2 Oncopathology Research Center, Iran University of Medical Sciences (IUMS), Hemmat Street (Highway), Next to Milad Tower, Tehran, 14496-14530 Iran; 3 Division of Nuclear Medicine, Department of Radiology, University of Iowa Carver College of Medicine, Iowa City, IA USA; 4 Division of Nuclear Medicine, Department of Radiology, Roswell Park Comprehensive Cancer Center, Buffalo, NY USA; 5 Department of Pathology and Genomic Medicine, Sidney Kimmel Cancer Center, Thomas Jefferson University, Philadelphia, PA USA

**Keywords:** Extracellular matrix (ECM), Matrix Stiffness, Mechanotransduction, Cancer stem cells (CSC), Metastasis, Chemoresistance

## Abstract

Matrix stiffness is a mechanical characteristic of the extracellular matrix (ECM) that increases from the tumor core to the tumor periphery in a gradient pattern in a variety of solid tumors and can promote proliferation, invasion, metastasis, drug resistance, and recurrence. Cancer stem cells (CSCs) are a rare subpopulation of tumor cells with self-renewal, asymmetric cell division, and differentiation capabilities. CSCs are thought to be responsible for metastasis, tumor recurrence, chemotherapy resistance, and consequently poor clinical outcomes. Evidence suggests that matrix stiffness can activate receptors and mechanosensor/mechanoregulator proteins such as integrin, FAK, and YAP, modulating the characteristics of tumor cells as well as CSCs through different molecular signaling pathways. A deeper understanding of the effect of matrix stiffness on CSCs characteristics could lead to development of innovative cancer therapies. In this review, we discuss how the stiffness of the ECM is sensed by the cells and how the cells respond to this environmental change as well as the effect of matrix stiffness on CSCs characteristics and also the key malignant processes such as proliferation and EMT. Then, we specifically focus on how increased matrix stiffness affects CSCs in breast, lung, liver, pancreatic, and colorectal cancers. We also discuss how the molecules responsible for increased matrix stiffness and the signaling pathways activated by the enhanced stiffness can be manipulated as a therapeutic strategy for cancer.

## Introduction

Solid tumors, as abnormal organs, are complex entities composed of heterogeneous populations of tumor cells and various types of stromal cells that produce soluble factors, signaling molecules, and extracellular matrix (ECM) components; which altogether can regulate tumor growth and progression and affect the response to treatment [1, 2]. The ECM is a three-dimensional network that mostly consists of macromolecules such as collagen, fibronectin, laminin, elastin, proteoglycans, and glycoproteins that provide structural and biochemical support to the cell [3]. During the progression of several solid tumors, deposition, remodeling, and crosslinking of the ECM composition alter and induce stiffening of the stroma from the tumor periphery to the tumor core in a gradient pattern [4, 5]. It has been demonstrated that high-grade invasive ductal carcinoma is 13-fold stiffer compared to normal human breast tissue [6]. Studies show that the stiffness of the ECM can effectively alter cell behavior at the cellular and molecular level through mechanosensing pathways [7]. Increased matrix stiffness appears to be a hallmark of solid tumor progression and metastasis. Considering the important function of the matrix stiffness in tumors, targeting the matrix stiffness has emerged as one of the next-generation therapies for cancer treatment [8, 9].

Cancer cells exhibit considerable heterogeneity in a variety of phenotypic and functional aspects [10]. Cancer stem cells (CSCs) are subpopulations of cancer cells that have similar characteristics to normal stem cells or progenitor cells. Recent findings suggest that cancer stem cells play a pivotal role in tumor initiation, progression, development, metastasis, resistance to treatment, and recurrence of cancer [11]. Also, there is evidence that the plasticity of tumors can lead to a dynamic variation in the relative abundance of CSCs and non-CSCs [12, 13]. Studies have shown that plasticity has significant implications for cancer therapies and cancers with a higher ratio of CSCs to non-CSCs are more resistant to chemotherapy [14]. On the other hand, after successful tumor resection, the remnant CSCs can lead to recurrence and be the culprit in certain forms of cancer cell dormancy, i.e. the state that the cells can remain dormant for many years, and suddenly awaking and causing overt recurrence and metastasis [15]. The interaction of non-CSCs with their surrounding microenvironmental niche contributes to their transformation into CSCs [16, 17].Among various components and signals of tumor stroma, matrix stiffness, arising from increased levels of collagen and enhanced crosslinking can have an impact on the formation, maintenance, and characteristics of CSCs[18, 19]. Higher matrix stiffness in tumors is associated with increased invasion and metastasis at least partly due to the increase in CSC population and markers [20, 21].

In this review, the effect of tumor matrix stiffness on some specific characteristics of CSCs including cell membrane CSC markers and tumor sphere formation (also used to enrich CSC/CSC like population) will be discussed. First, the molecular mechanisms by which stiffness of the matrix affects tumor cells are explored. Then effect of increased matrix stiffness on the progression and development of the tumor will be reviewed; and next, role of CSC in tumor progression, metastasis, drug resistance and recurrence will be explained. Following that, alteration in matrix stiffness in several solid tumors, including liver, breast, colorectal, lung and pancreas, and the effect of these changes on the special characteristics of CSCs will be reviewed. Finally, CSCs characteristics mediated by matrix stiffness alterations useful for obtaining novel insights into cancer biology will be discussed. Understanding how matrix stiffness regulates CSCs features and its functional consequences in cancer processes can represent a new perspective on cancer treatment.

## Molecular mechanisms by which increased matrix stiffness influences cell characteristics

**Fig. 1:**
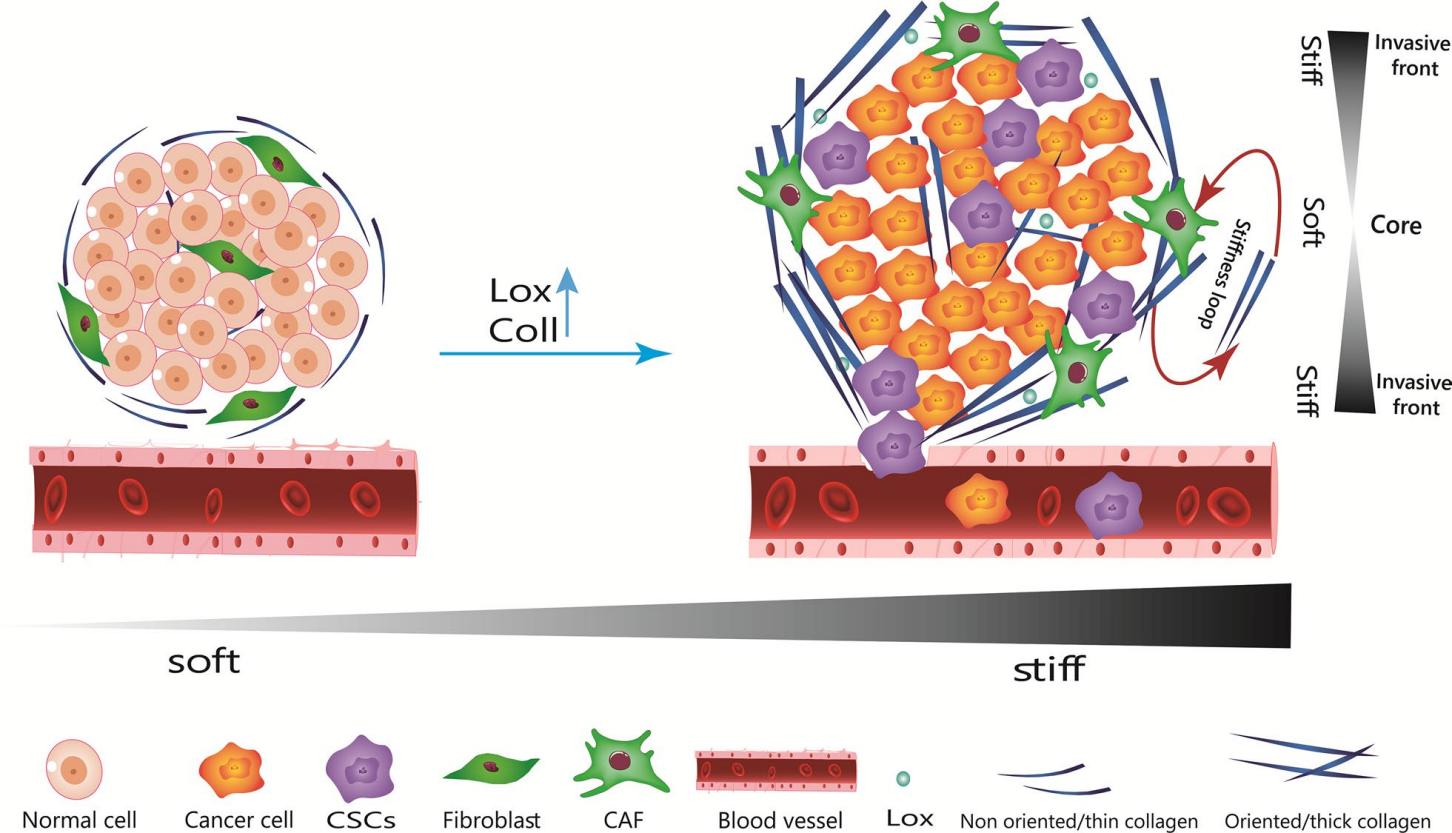
Alterations in tumor matrix stiffness: normal organs are surrounded by irregularly thin collagen, which forms an ECM that is compliant and soft. In several solid tumors, the accumulation of ECM proteins causes a gradual rise in matrix stiffness parallel with the tumor’s growth. Tumor cells and other TME cells, particularly cancer-associated fibroblasts (CAF), produce collagen and Lysyl oxidase (LOX), resulting in collagen crosslinking, ECM rearrangement, and increased stiffness. In addition, increasing stiffness within tumors contributes to the continuous activation of CAFs, establishing a feed-forward loop that aids in the formation of a permanently stiff tumor niche. It is important to note that CSCs are not distributed uniformly across cancerous tissues. More CSCs are distributed in invasive areas to facilitate metastasis. The invasive tumor front (ITF) is stiffer than the tumor’s core

In many solid tumors, concurrent with the progression of the tumor, the accumulation of several ECM proteins leads to a gradual increase in matrix stiffness and ECM rearrangement [4]. Tumor cells and other tumor microenvironment (TME) cells, especially cancer-associated fibroblasts (CAF), produce collagen, which forms the majority of the tumor matrix and enhance the production of Lysyl oxidase (LOX), leading to collagen crosslinking, ECM rearrangement, and higher stiffness [22, 23]. Also, increased stiffness within tumors contributes to the incremental and continuous activation of CAFs, establishing a feed-forward loop that aids to the development of a permanent stiff tumor niche [24] (Fig. 1). In advanced stages of breast and colon cancers, the expression of collagen I, LOX has been found to be significantly higher in the TME, resulting in increased stiffness [8, 25]. In normal breast tissue, collagen fibrils are relaxed and non-oriented, whereas in breast cancer these fibers are usually thicker and aligned [26]. Stiffness is defined as the resistance of a material to deformation when a force is applied [27]. Several techniques have been used to measure the stiffness of tissue, and stiffness values can differ significantly between methods (Table 1). In atomic force microscopy (AFM), a tip enters the specimen and the cantilever beam flexes in response to the sample's stiffness. By combining the tip position, cantilever spring constant, and piezoelectric sensor measurements, the stiffness of the tissue can be determined at a microscale [28]. A compression test based on specimen indentation and rheometry can measure macroscale (mm) stiffness [29]. Shear wave elastography (SWE), as a higher accuracy method, uses acoustic radiation to induce mechanical vibrations and measures the stiffness of a tissue by capturing propagating shear waves [30].

**Table 1:** Stiffness of human normal and tumoral tissues

Organs	Normal (kPa)	Tumor (kPa)	Methods of measurement	Ref.
Breast	3.25	Low-grade IDC: 10.40 DCIS: 16.38 High-grade IDC: 42.52	–	[6]
Lung	0.5–5	20–30	–	[74]
Liver^a^	–	55 in HCC 75 in CCC 66.5 in metastatic tumor	Transient elastography	[129]
Liver^a^	–	Low degree malignant: 8–15 High degree malignant: 14–18	AFM	[135]
Liver^a^	1.5–5	–	Shear elasticity	[134]
Pancreas^a^	0.4	1.2	AFM	[41]
Pancreas^a^	< 15	> 40	Harmonic motion elastography (HME)	[159]
Colorectal	0.9	Primary tumor (PT) stage T1: 2.8 T2: 3.5 T3: 8.8 T4: 13.8	Venustron system	[181]
		Distant metastasis Present: 13.6 Absent: 7		

^a^The reported stiffness may vary depending on measurement techniques

**Fig. 2:**
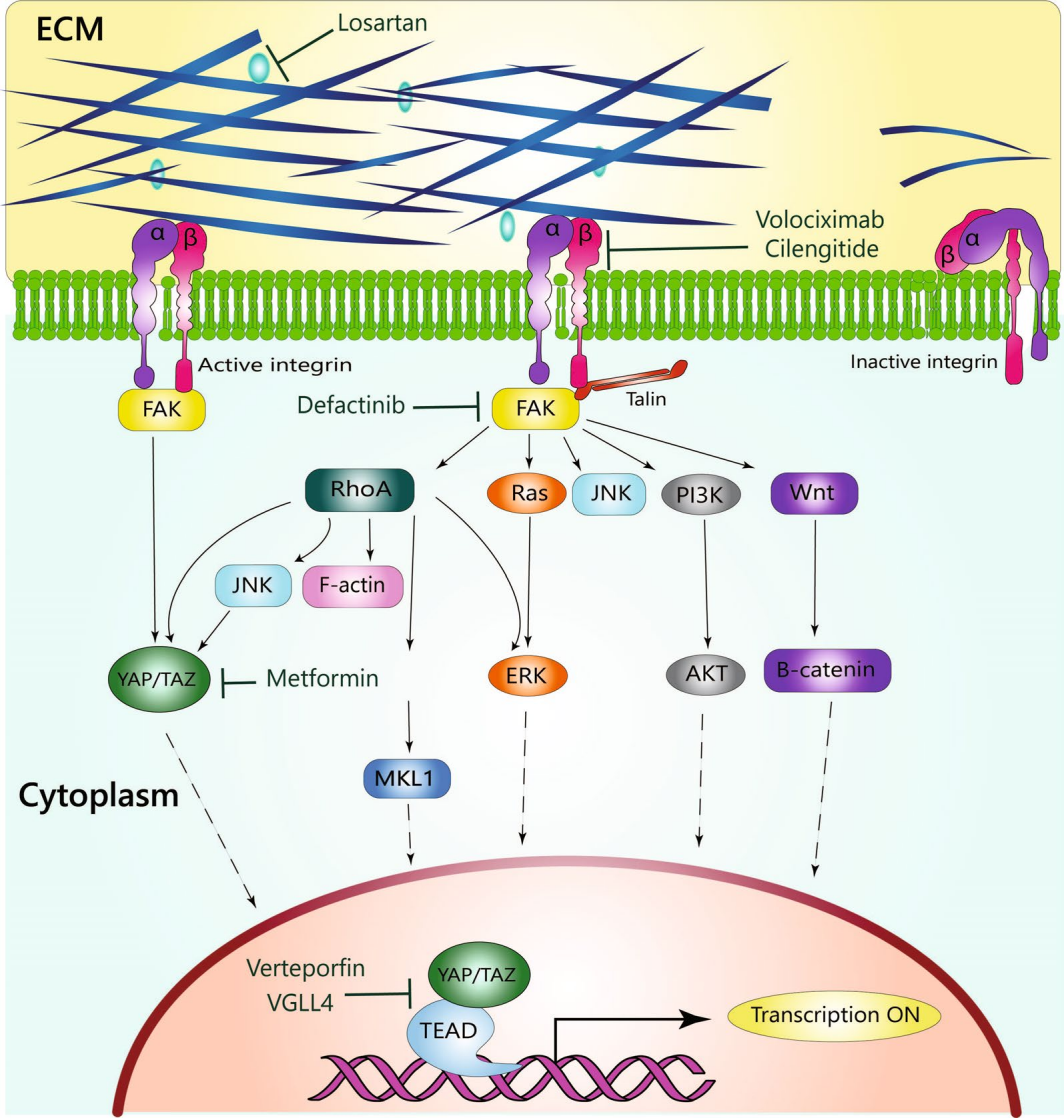
Matrix stiffness signaling pathways. Stiffness activates integrin-focal adhesion kinase (FAK) and activated FAK regulate several downstream mechanoresponsive signaling pathways. Pathways such as ERK, AKT, β-catenin, RhoA-ROCK, YAP/TAZ play major roles in stiffness mediated characteristics. In stiff ECM, YAP and TAZ are activated and accumulate in the nucleus, whereas in physiological stiffness, they are suppressed and localized in the cytoplasm. The transcriptional coactivators YAP and TAZ lack a DNA-binding domain. Hence, nuclear YAP/TAZ binds to TEAD and regulates the activation of several target genes involved in cell migration, proliferation, anti-apoptotic processes, and stemness

Through a process known as mechanosensing and mechanotransduction, tumor cells detect the stiffness index of the ECM and molecular effectors respond and transmit this signal, and then transform this information into biochemical signals that alter cellular behavior [31]. Integrin receptors respond to forces caused by increase in matrix stiffness. Each subunit of an integrin has a particular specificity for a particular ECM ligand, which can transmit the stiffness signal of the ECM into the cells via distinct mechanisms. Adapter molecules, such as focal kinase adhesion (FAK), accumulate in response to ligand binding to integrin receptors. The degree of matrix stiffness can regulate FAK activity and, consequently, the activation rate of several signaling pathways [32]. In these pathways, FAK can activate phosphatidylinositol-3-kinase (PI3K), serine/threonine-protein kinase (AKT), β-catenin, ERK, JNK, and other molecules, while inhibiting tumor suppressor genes such as phosphatase and tensin homolog (PTEN) and glycogen synthase kinase 3α/β (GSK3α/β) [33, 34]. Moreover, the cell can convey mechanical cues through the RhoA/Rho-associated protein kinase (ROCK) pathway [35]. Additionally, tumor stiffness influences tumor and stromal cells through the transcriptional activators yes-associated protein 1 (YAP1) and WW domain-containing transcription regulator 1 (WWDR1) (TAZ) [36]. In stiff environments, YAP and TAZ are activated and accumulate in the nucleus, whereas they are suppressed and localized in the cytoplasm in physiological stiffness [37]. YAP and TAZ are transcriptional coactivators lacking DNA-binding domain. Hence, these molecules must interact with DNA-binding transcription factors to regulate the expression of target genes. From there, based on the DNA-binding partner, tumorigenic and tumor suppressor genes can be expressed [38] (Fig. 2).

## The importance of matrix stiffness in cancer progression, development, recurrence and treatment

Multiple studies have demonstrated the importance of matrix stiffness in physiological and pathological states [19, 39]. Cell adhesion, migration, proliferation, and differentiation can be regulated by stiffness [39–41]. Matrix stiffness is important in embryonic morphogenesis and development [42]. During tissue injury and aging, alterations in matrix stiffness also play a special role [43] and significantly contributes to tissue homeostasis and function [39]. If the homeostasis of stiffness is disturbed for any reasons, it may lead to tissue dysfunction and the associated to pathologic conditions such as cancer [44].

**Fig. 3:**
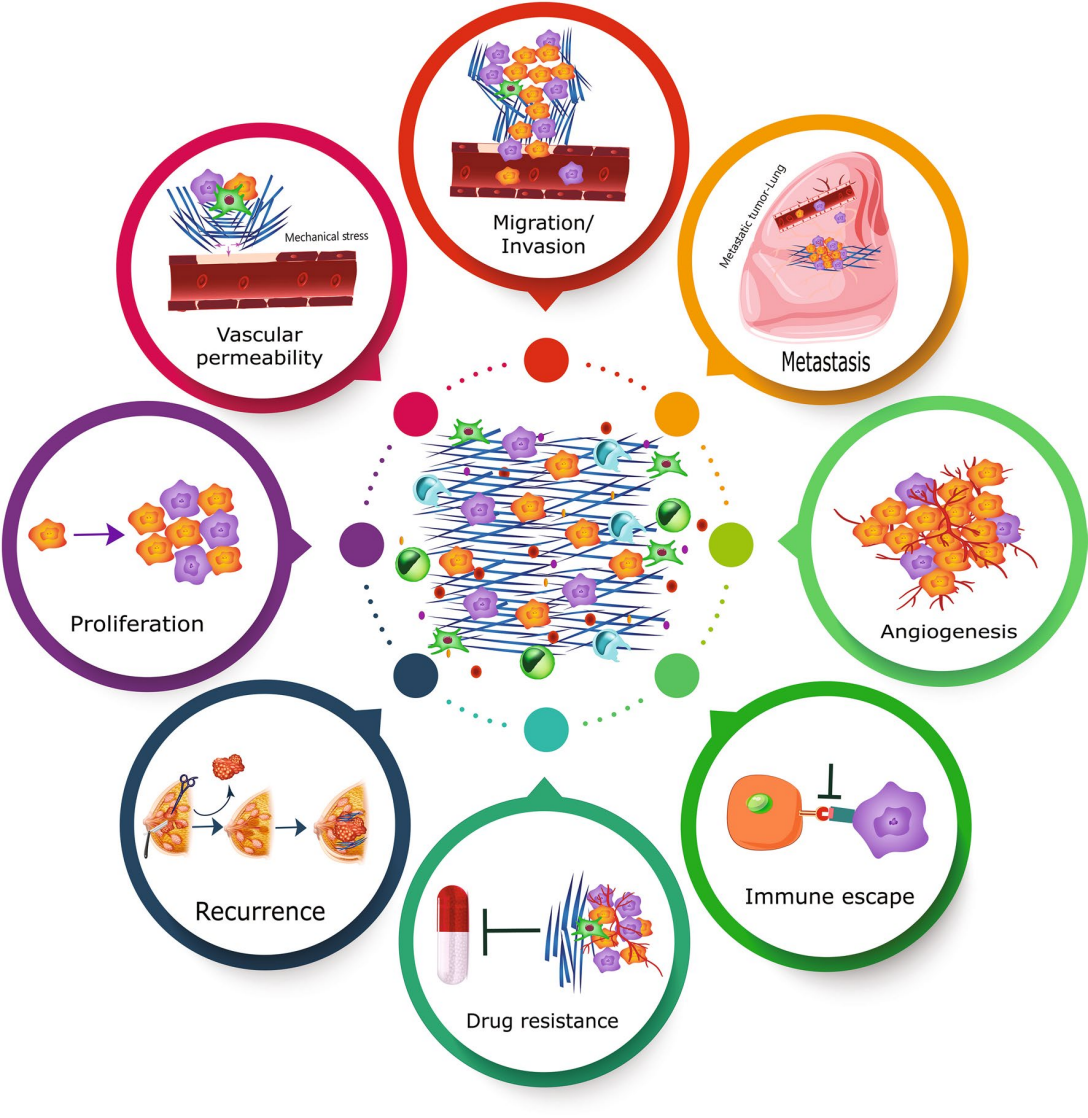
Several functions of increased matrix stiffness in cancer. Increased matrix stiffness can have an effect on tumor cell proliferation, vascular permeability, invasion and migration, metastasis, angiogenesis, immune evasion, treatment resistance, and recurrence

In a number of solid tumors, increased matrix stiffness can affect tumor progression, metastasis, and therapeutic response [31, 45] (Fig. 3). It’s important to note that the effect of tumor matrix stiffness on tumor progression and metastasis is a multifaceted phenomenon. Matrix stiffness in the primary tumor promotes the formation of CAFs, which in turn increases collagen and LOX synthesis, generating a positive feedback loop that promotes tumor progression and metastasis [45]. Interestingly, in terms of stiffness, tumors are heterogenous; for instance, in breast cancer, stiffness gradually increases from the core to the periphery [4, 46]. Different local maps of ECM stiffness could dictate distinct cancer cell functions during tumor progression. Breast cancer cells grown in a matrix similar to tumor's core stiffness exhibit increased proliferation, rise in glycolysis rate, and the high tumor formation potential; whereas, tumor cells grown in a matrix with variable stiffness that matched the peripheral zones of breast tumors show increased fibronectin 1 (FN1) and matrix metalloproteinase 9 (MMP9) expression, migration, oxidative phosphorylation (OXPHOS) and fatty acid (FA) metabolism processes, and also angiogenesis [4].

Tumorigenesis in breast is associated with ECM stiffening, stiffness induces the formation of integrin-FAK, leading to ROCK-generated contractility and promoting a malignant phenotype [47]. When cell–cell adhesions decreases due to the increased stiffness of the matrix, nuclear-activated YAP/TAZ binds to the TEAD and regulate the activation of several target genes involved in cell migration, proliferation, anti-apoptotic processes, and stemness [38, 48]. Most of the functions of YAP and TAZ include promoting sustained proliferation by expression of proto-oncogenes such as MYC and AP-1 family, transcriptionally upregulating the enzymes involved in metabolic requirements to support proliferating cancer cells [42], controlling the expression of cell cycle regulators, DNA replication and repair, and mitosis [49, 50]. YAP/TAZ can reprogram non-CSCs into CSCs [51]. This indicates that YAP/TAZ can modify the proportion of CSCs present in tumor tissues [52]. In addition, YAP is a critical molecule in the maintenance of CSCs in a variety of tumor types [53]. YAP/TAZ activation contributes to the induction of resistance to MAPK pathway–targeted therapies (RAF and MEK inhibitors) [54]. YAP/TAZ are involved in metastasis through several mechanisms, one of which is responsible for anoikis- resistance of circulating tumor cells (CTCs) [55, 56].The nuclear accumulation of YAP/TAZ can modify E/N cadherin and vimentin expression in response to stiffness, inducing epithelial–mesenchymal transition (EMT), a prerequisite for invasion and metastasis [57–59]. It is well known that the induction of EMT in carcinoma cells, produce stem cell-like cells [60], and would increase stem-like cell features [61]. Also, the stiffened ECM can exert a physical force on basement membrane to generate permeable pores, facilitating the invasion of CSCs [62].

Collagen fiber properties and organization (length, alignment, etc.), as stiffness characteristics can also be used as a prognostic marker and innovative paradigm for cancer metastasis and survival prediction [63, 64]. Enhanced stiffness -through a variety of mechanisms- can result in chemotherapeutic drug resistance [65–67]. Increased stiffness can establish a barrier and increase the interstitial fluid pressure within the tumor; thereby, it limits access, impairs perfusion and prevents drug delivery [68]. Matrix stiffness can induce EMT in pancreatic cancer cell lines and contribute to the role of EMT in chemotherapeutic drug resistance [41]. In a variety of cancers, stiffness serves as a predictor of chemotherapy response, so that the softer tumors are more drug-sensitive [69–71]. Measuring the stiffness of the liver prior to curative resection could be predictive of hepatocellular carcinoma (HCC) recurrence [72]. A stiffer matrix can upregulate osteopontin (OPN) expression in HCC cells through a Wnt-independent-β-catenin pathway (OPN is a molecule strongly associated with metastasis, early recurrence, and poor prognosis) [73]. EMT induction in immortalized human mammary epithelial cells results in the expression of stem cell markers [60]. This raises the possibility that the EMT-mediated stiffness established by surgery and scar formation may confer self-renewal capacity to epithelial cells, hence promoting cancer recurrence. Also, immune modulation is significantly affected by the matrix stiffness and the expression of programmed cell death-ligand 1 (PD-L1) [74]. The orientation, spacing, and density of collagen fibrils in the stroma can also affect the distribution and migration of CD8 T cells [75]. In addition to its effects on the primary tumor, matrix stiffness can influence the formation of the metastatic niche and the rate of metastasis [76–78]. Chu et al. revealed that matrix stiffness can regulate cellular adhesion and promote breast cancer cell homing in premetastatic niches [79] (Fig. 3).

Moreover, matrix stiffness indirectly affects tumor cell behavior through exosomes. Using stiffness-tunable scaffolds, Patwardhan et al. found that stiff ECMs promote exosome secretion in a YAP/TAZ pathway-dependent manner. Stiffness-mediated secreted exosomes promote cell motility and invasion. Based on genomic and proteomic profiling of secreted exosomes, thrombospondin-1 (THBS1) was identified as a regulator of tumor invasion, dependent on the stiffness of the tumor. THBS1 levels per exosome were significantly higher in stiff ECMs secreted exosomes, which were amplified by the greater total number of exosomes in stiff scaffolds. According to knockdown experiments, the pro-invasive effects of stiffness-tuned exosomes are driven by exosomal THBS1; MMP-9 and FAK are engaged by exosomal THBS1 in order to promote cancer invasiveness [80]. Therefore, stiffness-mediated secreted exosomes and their components can be used as potential therapeutics. As the effects of matrix stiffness on cancer processes is well known, comprehensive investigation of the effect of matrix stiffness on CSCs, one of the most crucial and challenging therapeutic targets, can lead to the development of novel cancer therapeutic strategies.

## The significance of cancer stem cells (CSCs)

Tumor cells are heterogeneous populations with significant differences in cell surface markers, gene expression, proliferation, invasiveness, and therapeutic response [81]. Two theories explain the heterogeneity of tumor cells: in the stochastic model, a unique population of tumor cells acquires mutations and develops the ability to metastasize. In the hierarchical model, a small subpopulation of cancer cells in a tumor adheres to a functional hierarchy, allowing for self-renewal and differentiation. In this model, CSCs are responsible for developing initial tumors and metastasis [82, 83]. Bonnet et al. reported in 1997 that acute myeloid leukemia (AML) contains a stem cell hierarchy that mimics the normal hematopoietic stem cell hierarchy. They revealed that serial transplantation of a rare population of CD34^+^ CD38^−^ leukemia cells can repopulate the tumor in its entirety; indicating that this population of cells possesses stem cell-like properties, such as the potential to proliferate. This research formed the basis for subsequent CSC studies [84]. CSCs are identified by stem cell markers such as CD44 + and CD133 + , and their characteristics are maintained via the expression of pluripotency factors such as Nanog and Oct-4 [11, 85, 86].

CSCs show a variety of characteristics, including self-renewal, proliferation, and differentiation into several cancer cell lineages through symmetric and asymmetric cell division, migration capacity, and specific surface markers. These cells are widely believed to play a critical role in tumor initiation, progression, development, metastasis, drug resistance, and recurrence [87]. Due to their increased chemoresistance and quiescence, CSCs are one of the most important cancer recurrence drivers [88]. The frequency of CSCs varies significantly between tumor types, ranging from < 1% in liver cancer to 82% in acute lymphoblastic leukemia (ALL) [89]. The proportion of CSCs in tumors is influenced by various parameters, including the host environmental conditions. The TME, especially the tumor stroma, is one of the parameters involved in the maintenance of CSC populations [90]. Moreover, changes in TME components can result in the dedifferentiation of mesenchymal or epithelial cells into CSCs [15]. Stem cell lineage commitment and differentiation can be affected by the stiffness of the matrix. In a soft matrix, mesenchymal stem cells (MSCs) differentiate into the neurogenic lineage, whereas in a stiff matrix, MSCs differentiate into the osteogenic lineage [91, 92]. Deregulation of ECM dynamics is essential for the formation of the niche for tumor stem cells and the generation of CSCs [93], and matrix stiffness as an important ECM characteristic can induce stemness [20]. Based on the CSC hierarchy/heterogeneity model and the importance of CSC, targeting all tumor cells with a similar approach would be ineffective, while eliminating the CSCs will eradicate the tumor and prevent recurrence. It is important to understand the matrix stiffness-mediated effects on CSCs in order to find targetable pathways that may be clinically advantageous. The next section discusses the effect of matrix stiffness on CSC in various cancers as one of the characteristics of the tumor stroma.

## Effect of matrix stiffness on the CSC population, characteristics, and functions various malignancies

### Effect of matrix stiffness on breast CSCs (BCSCs)

Breast cancer (BC) is the most common cancer in women and the fifth leading cause of cancer-related deaths worldwide [94]. Twelve percent of all women in the United States will be diagnosed with BC in their lifetime, according to the American Cancer Society [95]. Abnormal modifications in the quantity and organization of ECM components, such as collagen, occur during the progression of BC, leading to an increase in matrix stiffness that promotes tumor development and metastasis [32, 96]. Matrix stiffness is one of the most well-known risk factors for BC development [97]. Collagen fibers in grade 3 mammary carcinoma are thicker, longer, and straighter than those in grades I and II [98]. In BC patients, tumor stiffness can predict prognosis and classify treatment response [99]. Specific tumor-associated collagen signatures (TACS), which represent the density and organization of collagen fibers, can predict recurrence, therapeutic response, and clinical outcomes in BC [63, 97, 100]. These data highlight the importance of collagen organization and stiffness in breast cancer.

In the study by Samani et al. that evaluate the stiffness of normal and pathological human breast tissues, the elastic moduli of normal, low-grade invasive ductal carcinoma (IDC), ductal carcinoma in Situ (DCIS), and high-grade IDC were 3.25, 10.40, 16.38, and 42.52 kilopascal (kPa), respectively [6] (Table 1). The mean values of stiffness measured by SWE correlate with subtypes and histological characteristics. The ECM stiffness of ER-positive tumor cells was 136 kPa, HER2-positive 160 kPa, triple-negative BC (TNBC) 169 kPa, and the stiffnesses of grades I, II, and III were 117, 132, and 165 kPa, respectively [101].

Using the cell surface markers Epithelial Specific Antigen (ESA^+^), CD44^+^, and CD24^−^, populations of stem-cell-like cells in breast cancer can be identified [11, 102]. It is shown that in the xenograft mouse model, as few as 200 cells of these cells can form tumors [11]. In addition, the expression and activity of aldehyde dehydrogenase (ALDH) has been applied to isolate and detect human breast CSC populations. ALDH1^+^ CSCs are significantly aggressive; patients with ALDH1 positive tumor cells are more resistant to treatment, and prognosis is poor. ALDH1^high^ cancer cells generate more colonies and mammospheres than ALDH1^low^ cancer cells [103]. The stiffness of the matrix has a significant effect on the maintenance of BCSC phenotypes [19]. The translocation of YAP and TAZ to nuclear promotes the BCSC phenotype [52, 104], and TNBC has the highest proportion of cells expressing BCSC markers compared to other BC subtypes [105].

Increasing stiffness can lead to an increase in BCSC as shown in both *in-vitro* and *in-vivo* studies. Application of mechanical forces can increase the CSC populations in MCF-7 breast cancer cells [106]. In 3D scaffolds with the same stiffness as breast tumor tissue, stemness markers (Nanog, Sox2, and Oct4) and CD44 were found to be expressed at a higher level than in 2D cultures. Also, sphere formation was higher in the scaffold than in 2D culture, indicating an increase in stemness and metastatic potential [107]. In aligned collagen matrices, the motility and contact-guided migration of BCSCs were significantly enhanced [108]. Using polyacrylamide (PA) substrates to mimic the stiff (4020 pa) and soft (120 pa) microenvironments of breast tumors and normal tissues, respectively; in the stiffer matrix, 4T1 and MDA-MB-231 cells expressed higher CSC markers, including CD44, Nanog, CD49, and ALDH and in regions of human breast cancer with low collagen levels (soft region), only 4% of tumor cells expressed CD44 and Integrin-linked kinase (ILK), a crucial mediator used by cells to sense their surroundings as opposed to the regions of BC with high collagen levels, where more than 25% of cells expressed CD44 and ILK. These results indicate that breast CSCs are frequently found in the dense regions. In 4T1 and MDA-MB-231 cells cultured on stiff substrata, ILK knockdown reduced CSC markers and decreased the tumorigenic and metastatic potential of tumors [19]. Following culturing human MCF7 and MDA-MB-231 breast cancer cells on Polyethylene Glycol Diacrylate (PEGDA) gels with moduli ranging from 2 to 70 kPa, it was found that 5 kPa was the optimal stiffness for maintaining the population of BCSCs [109]. When 4T1 breast cancer cells were grown on PEGDA hydrogels, increasing matrix stiffness from 2.5 kPa to 5.3, 26.1, and 47.1 kPa, resulted in tumor sphere size increasing from 37 to 57 µm, 20 µm, and 12 µm, respectively, and CD44 expression increased from 17-fold to 38-fold, threefold, and twofold, respectively, compared to the baseline levels. Additionally, MCF7 human breast cancer cells had similar results, and cells cultured in gel with modulus of 5.3 kPa showed the highest CD44 expression and the largest tumor spheres [110] (Table 2).

**Table 2:** Effect of matrix stiffness on CSCs population and characteristics

Cancer	Cell type	Material of scaffold	Stiffness	CSC markers/stemness-related genes and genes affecting CSCs	Methods of investigation	Results	Ref.
Breast	4T1, MCF-7	polyethylene glycol diacrylate hydrogels (PEGDA)	2.5, 5.3, 26.1, and 47.1 kPa	ABCG2, CD44	qRT-PCR	5,3 kPa was the optimal stiffness for higher tumorsphere size and CSCS marker expression	[110]
	MCF-7, MDA-MB-231	PEGDA	2–70 kPa	CD44	qRT‐PCR, Flow-cytometry	5 kPa is the optimum matrix stiffness for BCSC growth and CSC marker expression	[109]
	MDA-MB-231	Polycaprolactone (PCL)	3D: 7 kPa and 2D	CD44, Nanog, Sox2, Oct4	q-PCR	7 kPa-cultured cells express higher levels of the stemness marker CD44	[107]
	MDA-MB-231	GelMA/collagen	2 and 12 kPa	MENA	qRT-PCR	Stiff matrix-grown cells express more MENA, a breast cancer metastasis-associated invadopodia protein. MENA overexpression increases the cancer stem cell-like phenotype	[111]
	MDA-MB-231, 4T1	Polyacrylamide (PA)/collagen	130, 4020 Pa	CD44, Nanog, CD49f, ALDH	qRT-PCR, Immunofluorescence	25% of cells in the stiff scaffold and 4% in the soft scaffold expressed CD44	[19]
Lung	A549	PA crosslinked with bisacrylamide	0.2, 2, and 25 kPa	c-Met, EGFR, snail	RT-PCR, Western blot	With increasing matrix stiffness, EGFR and c-Met expression increased, causing tumor cell proliferation and resistance to EGFR and c-Met inhibitors	[124]
	A549, NCI-H1395, NCI-H1650, and PC9	2D 3D: (very stiff: ABS, HIPS, PLA) and stiffness comparable to lung cancer (GelMA-PEGDA)	> 90 M pa < 0.01Mpa	several stem markers such as ALDH1A1, NANOG, CD44, …	qRT-PCR	More CSC markers were expressed by cells cultured on scaffolds than in 2D culture. Less CSC markers were expressed by cells seeded on ABS, HIPS, and PLA scaffolds than on GelMA-PEG scaffolds	[126]
	H1299	Matrigel coated with collagen	0.5, 4 and 25 kPa	CD133, NANOG	Immunoblotting, IHC, RT-PCR	H1299 cells cultured in a 4 kPa scaffold showed higher CD133 and NANOG expression than cells cultured in a 0.5 or 25 kPa scaffold	[20]
Liver	MHCC97H, Hep3B, and HepG2, Huh7	Matrigel	1 to 40 kPa	EpCAM, CK7, CK19	qRT-PCR	Higher expression in cells cultured in medium stiffness	[144]
	Huh7, HepG2	PA coated with collagen	1–12 kPa	OCT4, NANOG, c-kit, CD44, CXCR4, CD133	qRT-PCR	CSCs' marker expression was higher in cells cultured on a softer substrate	[150]
	Huh7, Hep3B	PA coated with collagen	6, 10, and 16 kPa	EpCAM, CD133, Nanog, SOX2	qRT-PCR, Flow cytometry	In the stiffer matrix, cells express more stemness-related genes, including SOX2 and Nanog, have high self-renewal capacity, and are CD133( +)/EpCAM	[143]
	Hep3B, Huh7	PA coated with collagen	1, 6 and 12 kPa	EpCAM, CD133, ALDH-1, CXCR4 and EMT Marker	qRT-PCR, Western blot, IHC	CXCR4, N-cadherin, and vimentin are upregulated in cells with a stiffer matrix, while E-cadherin is downregulated. The levels of EpCAM, CD133, and ALDH-1 were higher in stiff gels than in soft gels	[151]
	MHCC-97H	PA	0.4 to 25.6 kPa	miR-3682-3p	qRT-PCR	Stiffness of 25.6 kPa can enhance miR-3682-3p. Upregulation of MiR-3682-3p increased HCC spheroid formation, side population cell fractions, and CSC marker expression	[147]
	SMMC-7721, HepG2	PA	12, 16 kPa	CD133	Flow cytometry	Increased colony number and CD133 expression in stiff scaffolding	[135]
PDAC	PDAC organoids	HELP (Hyaluronan and elastin-like protein) Low, or HELP High matrices	279, 1253, and 3040 pa,	CD44, ABCG2, and CD24	Flow cytometry	The levels of CD44, ABCG2, and CD24 were higher in HELP High matrices. Moreover, significantly more side populations (SP) were seen in organoids grown in a high matrix (3.74%) than in a low matrix (0.74%)	[171]
Colorectal cancer	HCT-8	polystyrene	3.6 GPa	ALDH3A1, TNS4, CLDN2, and AKR1B10	qRT-PCR, Immunofluorescent	TNS4, CLDN2, AKR1B10, and ALDH1A1 are upregulated on scaffolds at 20 kPa	[174]
		PA	1–20 kPa				
	CCD18 (colon fibroblast cells)	fibronectin-functionalized PA	2, 10, 40, 95, and 120 kPA	activin A	ELISA	At 40 kPa, the level of Activin A in the conditioned medium of CCD18 cells reached a plateau. Activin A promoted invasive ALDHhi CSC-like phenotypes, cancer cell plasticity, and metastatic potential	[190]
	HCT‐116	collagen‐coated PA	2 to 20 kPa	CD133, ALDH‐1, Lgr‐5	qRT‐PCR, Western blots	HCT116 cells on stiff scaffold displayed higher expression of CD133, ALDH1, and Lgr5, as well as YAP nucleation	[186]
	HCT-116	PEGDA	2–70 kPa	CD44	qRT‐PCR, Flow-cytometry	25 kPa is the optimal matrix stiffness for HCT116 tumor stem cell growth and marker expression	[109]

**Fig. 4:**
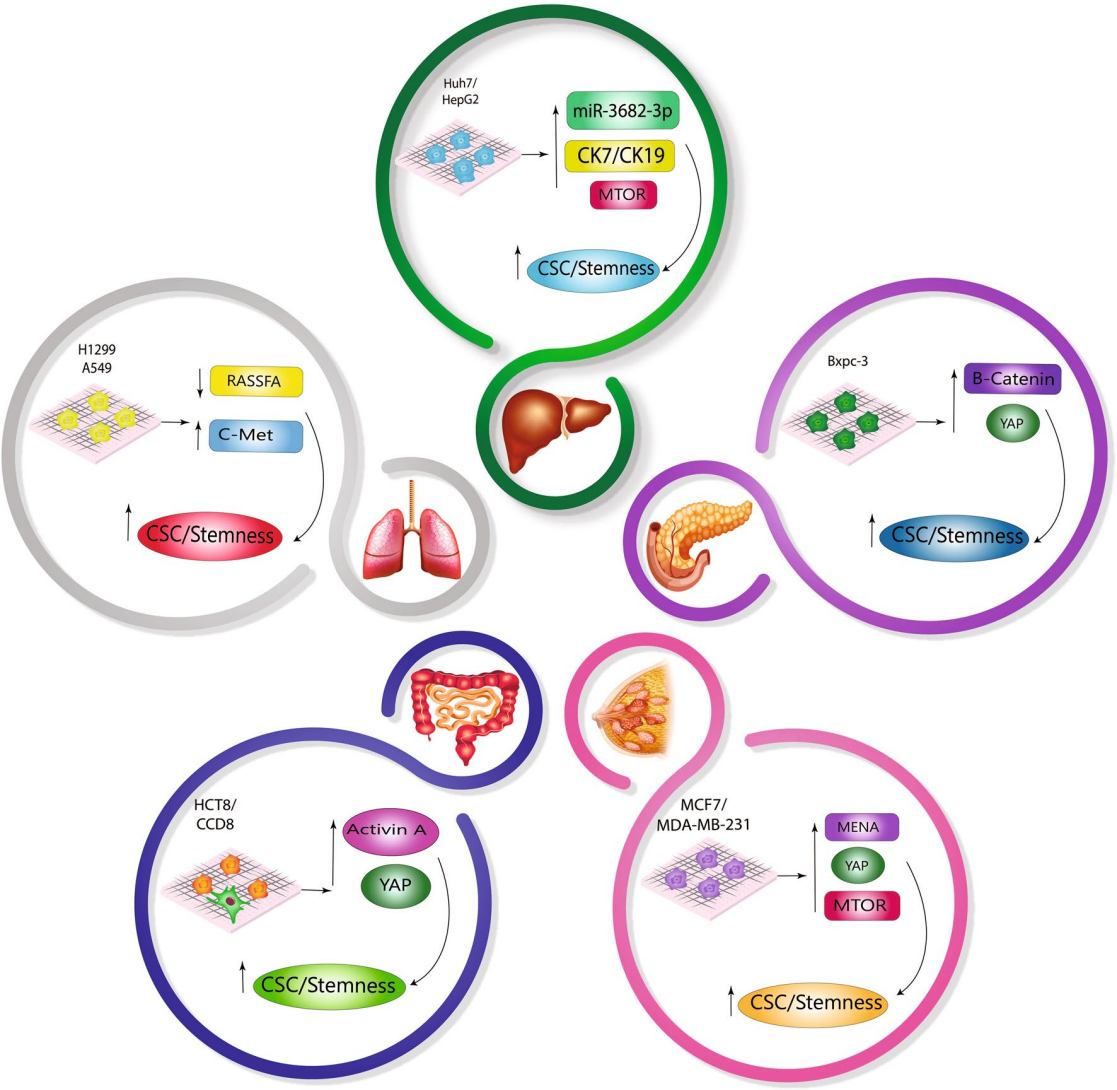
The effect of matrix stiffness on the CSC population in several malignancies. Molecular expression and activation were altered when breast, lung, liver, pancreatic, and colon cancer cell lines were cultured on a scaffold with gradient stiffness. These molecules enhance CSC population and stemness characteristics

Following culturing the MDA-MB 231 breast tumor spheroids on 3D hydrogels with 2 kPa and 12 kPa stiffness, in cells cultured in a stiff environment, the expression of Mammalian-enabled (MENA), an invadopodia protein associated with breast cancer metastasis, was observed [111] (Fig. 4). Overexpression of MENA in cancer cells could increase CSC production and EMT markers expression [112]. Accumulation of Collagen I in tumor of Col1a1^tmJae/+^ mice and higher CSC activity due to AKT-mTOR and YAP activation is shown, and that these mice have more and larger lung metastases. Rapamycin, an inhibitor of mTOR, decreased mammary tumor size and CSC activity. However, in contrast to primary tumor, inhibition of mTOR signaling did not inhibit lung metastases due to the lower activity of mTOR and proliferation activity in lung cells compared to mammary tumors; as a result, Col1a1^tmJae/+^ mice continued to sustain higher metastatic burdens. These findings shed light on the association between stiffness and CSC activity and metastatic behavior; it also emphasizes the different therapeutic responses of local versus distant breast cancer lesions [113]. As such, increasing stiffness in breast cancer leads to higher BCSC rates and modified CSC features, which promote aggressive behaviors and metastasis in BC.

### Effect of matrix stiffness on lung CSCs (LuCSCs)

Lung cancer is the leading cause of cancer-related death (18% of mortality) and the second most commonly diagnosed cancer [94]. According to one study,lung tumors are more stiff (20–30 kPa) than normal lung parenchyma (0.5–5 kPa) [74] (Table 1). In another study, the matrix stiffness of healthy lung parenchyma was reported to be 0.15 to 0.2 kPa, whereas fibrotic lung parenchyma had a matrix stiffness of 15 kPa [114]. Injury-repair and tumorigenesis are associated, and injury-induced inflammation can result in lung fibrosis, and a stiff tissue matrix increases the risk of carcinogenesis [114]. Non-small-cell lung cancer (NSCLC) patients with lung fibrosis had a worse prognosis and treatment response rate than NSCLC patients without lung fibrosis [114]. Increased collagen expression in NSCLCs activates FAK and ERK signaling pathways and promotes cytokine production such a IL-23, hence promoting lung cancer progression [115]. Moreover, primary tumor resection develops hypoxic areas that are a source of LOX, which enters the circulation and ultimately reaches to the lungs, resulting in a stiffer environment that promotes lung metastasis through FAK activation [116]. Due to FAK activation and collagen-dependent metastasis, FAK inhibitors may improve survival [116, 117]. Downregulation of miR-29a is associated with posttranslational overexpression of LOXL2 in lung cancer, promoting tumor progression through modulating ECM stiffness [118].

In addition, the increased stiffness of lung can affect immunomodulation. Expression of programmed cell death-ligand1 (PD-L1) on cancer cells is important for immune evasion. and it is also positively correlated with EMT, cell migration, and invasion [119]. Multiple mechanisms, including matrix stiffness, regulate PD-L1 expression. When HCC827 lung cancer cells were grown on 2 and 25 kPa PA hydrogels; on substrates with higher stiffness, PD-L1 protein expression was higher than in the 2 kPa gel [74]. The YAP/TEAD complex regulates PD-L1 transcription by binding to the PD-L1 promoter. Due to YAP overexpression, PC9 adenocarcinoma cells express PD-L1 at a higher level [120]. Interestingly, CSCs and PD-L1 are correlated. A positive association between CD44 and PD-L1 expression in lung adenocarcinoma patients is shown [121]. In the context of lung tumors, stiffness can regulate the expression of PD-L1 in CSCs, hence facilitating immune evasion and tumor growth.

Several markers, including ALDH, ATP binding cassette subfamily G member 2 (ABCG2), CD44, CD117/KIT, CD133, and stem markers Nanog and OCT3/4, are overexpressed in lung cancer and have been used to identify CSC populations [122]. Malignant lung tissues with stiffer matrix are more favorable for CSC formation and maintenance via YAP/TAZ signaling pathways [20, 123].

Following is a series of studies that evaluate the effect of matrix stiffness on LuCSCs. A549 cells were seeded on matrices with stiffnesses of 0.2, 2, and 25 kPa to mimic physiological, fibrotic, and severe fibrosis tissues, respectively. Increasing stiffness elevated the expression of epidermal growth factor receptor (EGFR) and hepatic growth factor receptor (c-Met), leading to an increase in tumor cell proliferation and EMT [124].Khater et al. found that c-Met signal transduction increases bulk tumor CSC enrichment and self-renewal potential [125]. In another study, several lung cancer cell lines were cultured on 2D, very stiff scaffolds (ABS, HIPS, and PLA) and stiffness comparable to lung cancer (GelMA-PEGDA). The levels of CSC markers expression in cells grown on scaffolds were significantly higher than in 2D culture. However, the expression levels of cells seeded on very stiff scaffolds (ABS, HIPS, and PLA) were lower than those on GelMA-PEG-based scaffolds [126].

In an elegant study, Pankova et al. used two cells: 1- H1299 cell line that is highly methylated and lacks the expression of RASSF1A as control and 2- H1299 that continuously express RASSF1A. Both cells are seeded on collagen-coated matrigel with defined stiffness. Soft ECM (0.5 kPa) induces the reprogramming of H1299^control^ cells to a cancer stem cell-like state and NANOG expression, but not in H1299^RASSF1A^ cells. Increasing the ECM stiffness (4 kPa) enhanced NANOG expression in H1299^RASSF1A^ cells. These data support the correlation between cancer stemness and ECM stiffness and suggest that RASSF1A suppresses stemness in soft ECM. Surprisingly, the expression of NANOG and CD133 was not increased in H1299^control^ and H1299^RASSF1A^ cells grown on a very stiff (25 kPa) scaffold [20]. They concluded that high matrix stiffness may lock the ECM conformation, preventing the exposure of binding sites such as integrins and so decreasing the capacity to respond to ECM [127] (Table 2). Moreover, IHC staining of H1299^control^ primary tumors showed NANOG expression and significant levels of nuclear YAP1, whereas in H1299^RASSF1A^ tumors, the majority of YAP1 was localized in the cytoplasm and with is no detectable NANOG staining [20]. These results support the stiffness-mediated activation of NANOG and cancer stemness. Accordingly, increased lung cancer stiffness correlates with higher lung CSC fraction and modified CSC characteristics, which promotes aggressive behaviors and metastasis.

### Effect of matrix stiffness on liver CSCs (LCSCs)

Liver cancer is the third leading cause of cancer-related deaths worldwide [94]. Approximately 90% of liver cancers are HCC and 10% cholangiocellular carcinoma (CCC) [128]. Liver stiffness increases in primary and metastatic cancers and promotes proliferation and cancer development [129]. Over 80% of patients with HCC have a background of cirrhosis or severe liver fibrosis [130, 131]. There is a two-to fivefold increase in total collagen content in a cirrhotic liver, and an increase in type I collagen is the primary distinguishing hallmark of liver fibrosis [132]. Importantly, fibrosis precedes the development of HCC, making it an important characteristic of the premalignant hepatic milieu. It is estimated that approximately one-third of cirrhotic patients will eventually develop HCC [24]. Patients with chronic hepatitis B with liver stiffness greater than 13 kPa had a 4-to 13-fold increased risk of HCC. In one study, it was shown that no patient with liver stiffness < 12 kPa had HCC within 21.8 months of follow-up, whereas 26% of those with liver stiffness > 12 kPa developed HCC. [133]. Thus, increased matrix stiffness in HCC promotes tumor progression and metastasis. Research on HCC indicates that matrix stiffness can modulate cell proliferation, angiogenesis, metastasis, and drug resistance [24].

Using a shear elasticity probe, the elastic modulus of a healthy liver ranged between 1.5 and 5 kPa, but it ranged between 5 and 69 kPa for fibrosis grades 1 to 4 [134]. According to one report, the tissue stiffness for HCC, CCC and metastatic tumors are 55, 75 and, 66.5 kPa respectively [129]. AFM analysis of HCC tissue stiffness classified patients into low degree (8–15 kPa) and high degree (14–18 kPa) malignant groups [135] (Table 1). The liver stiffness, as measured by Two-Dimensional Shear-Wave Elastography, could be an effective predictor of overall survival (OS) following radiofrequency ablation (RFA) for HCC. Patients with stiffness ≥ 13.3 kPa had a 3-year OS of 76.8%, whereas patients with stiffness < 13.3 kPa had a 3-year OS of 96.3% [136], and a later study confirmed these results [72]. In addition, liver cancer displays mechanical heterogeneity, with the invasive tumor front (ITF) becoming stiffer than the tumor's core. Intriguingly, the distribution of LCSCs correlates with the stiffness of the tumor, with the highest proportion of these cells be observed at the ITF [5].

LCSCs are identified by surface markers including CD133, epithelial cell adhesion molecule (EpCAM), CD90, CD44, CD24, CD13, OV6, and ALDH activity. LCSCs are associated with increased proliferation, tumorigenicity, metastasis, radiation or chemotherapy resistance, recurrence, and poor prognosis [137, 138]. EpCAM-positive HCC cells show highly tumorigenic capacity and CD90-positive HCC cells are highly metastatic [139]. It has been shown that chronic inflammation-induced stiffness increases the overall population of HCC stem cells [140]. Tumor-associated macrophages (TAM) are the cells involved in this phenomenon. Both M1 and M2 macrophages TAMs contribute to the synthesis of ECM molecules [141], and M2 can support stem cells and regulate their behavior as a part of the niche. Considering the increased stiffness of the matrix in HCC and the significance of CSCs, here we review a number of studies on the impact of matrix stiffness on LCSCs.

Culturing SMMC-7721 cells on stiff PA hydrogels increased the expression of stemness genes [135]. The addition of matrigel, collagen 1, or methyl cellulose to the sphere-forming culture medium significantly increased the initial oncosphere formation and the expression of pluripotent and stemness markers in LCSCs in response to increased stiffness [142]. Huh7 and Hep3B cells were cultured on COLI-coated PA gel substrates with tunable stiffness (6, 10, and 16 kPa). In the stiffer matrix, cells display increased stemness-related gene expression, including SOX2 and Nanog, as well as with high self-renewal capacity and a high proportion of CD133^(+)^/EpCAM cells. In addition, the phosphorylation levels of AKT and mTOR are increased in cells on the stiffer matrix. The knockdown of integrin beta 1 reduces the phosphorylation of AKT and mTOR molecules, hence decreasing the cellular response to stiffness. Moreover, mTOR inhibitors decrease SOX2 expression; thus, stiffness may exert its effect on cells through the integrin beta 1 molecule, and with the cascade of events such as phosphorylation and activation of the molecular pathways of AKT and mTOR, ultimately leads to increased expression of the stemness genes [143]. In a related study, several human HCC cell lines, including MHCC97H, Hep3B, HepG2, and Huh7, were cultured on gels of three distinct stiffnesses (from 1 to 40 kPa). In medium stiffness-cultured cells, EpCAM and cholangiocyte markers, including cytokeratin7 (CK7) and CK19, were considerably increased. The formation of tumors and the expression levels of EpCAM, CK7, and CK19 were also elevated in mice injected with cells derived from medium-stiffness gels [144]. The presence of CK, a stem cell marker, is associated with a poor prognosis [145, 146]. Also, miR-3682-3p was significantly up-regulated in stiffness-cultured MHCC-97H [147]. In HCC cells, the upregulation of miR-3682-3p improved the spheroid forming capacity, the side population cell fractions, the expression of CSC factors [148], and the poor prognosis in HCC patients [149]. Contrary to previous studies, the culture of Huh7 and HepG2 cells on a PA coated with collagen-I showed that soft substrate cells expressed higher levels of CSCs markers, including OCT4, Nanog, CD44, CD133, and CXCR4 as a chemokine receptor. Due to the use of a soft matrix with a stiffness of 1 kPa, which does not accurately represent hepatic stiffness in normal or pathological livers, the results of this study may be contradictory [150] (Table 2).

In a recent study, Yang et al. [151] found that as matrix stiffness increased, CXCR4 expression in HCC cells increased significantly, promoting EMT and stemness. According to the known role of CXCR4 on CSCs function, it was determined that increased expression of CXCR4 correlates with chemotaxis, invasion, and CSC characteristics in a variety of solid tumor malignancies, and that treatment with miR-139, which directly targets CXCR4, inhibited mesenchymal traits of CSCs [152, 153]. Matrix stiffness acts through CXCR4 to decrease the levels of ubiquitin domain-containing protein) UBTD1(, which is involved in the degradation of YAP, hence activating YAP-targeted genes and YAP downstream signaling [151]. In addition, activated metastasis-associated fibroblasts increase liver stiffness and promote angiogenesis, thereby providing sufficient nutrients for CSCs. Due to ECM stiffness, colorectal cancer patients with liver metastases are resistant to anti-angiogenic therapy. Inhibitors of fibroblast contraction reduce metastatic liver stiffness and increase bevacizumab's antiangiogenic effects [154]. In conclusion, recent research indicates that increasing stiffness can elevate the population of LCSCs and amplify their characteristics.

### Effect of matrix stiffness on pancreatic CSCs (PaCSCs)

Pancreatic cancer is an aggressive malignancy that counts as the seventh leading cause of cancer-related death worldwide [155]. It has one of the worst prognoses among solid tumors, with a 5-year survival rate of less than 10% [155, 156]. Despite increased understanding of pancreatic cancer risk factors and the development of new diagnostic techniques, the incidence of pancreatic cancer is still increasing. Pancreatic ductal adenocarcinoma (PDAC) is estimated to become the second leading cause of death from cancer by 2030 [157]. TME is very prominent in PDAC, and approximately 90% of the tumor volume is composed of stromal cells and extensive ECM deposition [158]. The elastic modulus of the non-neoplastic adjacent pancreas was less than 15 kPa, whereas PDAC tumors was over 40 kPa, measured by Harmonic Motion Elastography (HME) [159]. Also, AFM analysis revealed that the stiffness of normal pancreatic tissue was 0.4 kPa and that of pancreatic cancer tissue was 1.2 kPa [41] (Table 1). Pancreatic cancer tissues had higher amounts of collagen, hyaluronan, and the CD44 receptor. In addition, the transition from a healthy pancreas to invasive pancreatic ductal adenocarcinoma is accompanied by an increase in the thickness of collagen fibers, which is associated with a poor prognosis [160]. A high strain ratio (SR), which reflects the stiffness of pancreatic tissue, has prognostic value, and as higher SR predicts poor overall survival [161].

Increased collagen I expression in PDAC is associated with higher invadopodia formation in invading cancer cells, increased metastasis, and poor prognosis [162]. Also, stiffness can affect the sensitivity of pancreatic cancer cells to chemotherapy [68]. More than 90% of PDAC patients have mutant oncogenic KRAS, which is activated and converges with downstream signaling pathways such as YAP/TAZ [163]. Zhang et al. found that YAP is essential for tumorigenesis and the development of invasive PDAC in mice inoculated with KRAS mutant neoplastic pancreatic ductal cells [163]. Also, activation of the YAP1/TEAD complex cooperatively acts to promote PDAC recurrence in the absence of oncogenic KRAS, implying a novel mechanism for PDAC recurrence independent of the KRAS mutation. This suggests that YAP1/TAZ-dependent signaling may be essential for the early development and recurrence of PDAC [164].

Less than 1% of pancreatic tumor cells are CSCs, and the elimination of PaCSCs is a necessity for any PDAC therapeutic treatment [165]. Several cell-surface markers are used to detecting pancreatic CSCs. CD133, CD24, CD44, EPCAM, ESA, c-Met, Aldh1, ABCG2, and more recently, DclK1 and Lgr5 have been identified as markers of PaCSCs [166]. In pancreatic cancer, stiffness-sensing receptors activate Ras, Rac, MAPK, and PI3K signaling pathways, resulting in increased cell proliferation and stem cell characteristics [167]

In a recent study, 1, 4, and 25 kPa of acrylamide/bisacrylamide were utilized to determine the impact of stiffness on pancreatic cancer cells. The results demonstrated that BxPC-3 cells are more resistant to chemotherapy when cultured on matrix with 4 and 25 kPa stiffness. In a mouse model, stiff pancreatic cancer tissues led to EMT, increased vimentin expression, decreased E-cadherin expression, treatment resistance, and increased β-catenin and YAP nucleus localization [41]. In response to the increased matrix stiffness, YAP increases CD133 expression, which leads to an increase in cell proliferation and metastasis [168, 169]. Moreover, it has been shown that pancreatic tumors with a high proportion of tumor-associated fibroblasts (TAFs) have more drug-resistant and stem-like cells due to the fact that TAFs increase ECM synthesis in response to inflammation [170].

PDAC organoids were expanded on the HELP (Hyaluronan and elastin-like protein) Low and HELP High matrices with stiffnesses of 279, 1253, and 3040 Pa, respectively, to determine the effect of matrix stiffness on CSCs. In comparison to HELP Low, CSC markers such as CD44, ABCG2, and CD24 increased in HELP High matrices. CSCs frequently exhibit chemo resistance through the altered expression of drug transporters. The drug efflux transporters (ABCG2, ABCC3/4/5) associated with PDAC chemoresistance are increased in organoids grown on stiff matrices [171]. Also, the side population (SP), which has become an important hallmark for defining the stem-cell population [172], was significantly larger in organoids grown in the high stiff matrix (3.74%) than in the low stiff matrix (0.79%). Intriguingly, PDAC organoids that expanded in the stiff matrix were not drug-sensitized, but when switched to a matrix with low stiffness, they became drug-sensitized. Also, following multiple passages, the expression of CD44 and ABCG2 decreased in the soft matrix [171] (Table 2). These findings indicate that stiffness can affect PaCSCs and suggest that treatment of PDAC tumors with drugs that target matrix stiffness in combination with anti-cancer agents may improve therapeutic sensitivity of tumors, reduce the aggressive behavior of CSCs, and improve patient outcomes.

### Effect of matrix stiffness on colorectal CSCs (CCSCs)

Colorectal cancer (CRC) is the second leading cause of cancer-related deaths and ranks third in incidence worldwide [94]. In 2021, it is estimated that there were approximately 149,500 new cases and 52,980 deaths in the United States due to CRC [173]. CRC tissue is stiffer than normal tissue and promotes the proliferation, invasion, and metastasis of CRC cells [174, 175]. Recent research found that in the regions 10 to 20 cm away from the tumor, the ECM of uninvolved rectal mucosa is remodeled and stiffness is increased. Hence, the fact that increased matrix stiffness in CRC is not restricted to the primary lesion shows that the effect of increased matrix stiffness in CRC is very complicated [176]. Positive correlation between YAP/TAZ expression and poor prognosis in CRC patients emphasizes the carcinogenic properties of mechanoregulators in CRCs [177, 178]. YAP also promotes CRC chemotherapy resistance and cancer recurrence [179]. YAP inhibition reduced CRC cell lines proliferation and metastasis considerably, whereas YAP overexpression enhanced the rate of cell proliferation [180].

Correlation of CRC tissue stiffness with the clinicopathological characteristics of patients were evaluated by Kawano et al. The median elastic modulus (EM) of normal colorectal tissue was 0.90 kPa, which is considerably lower than the median EM of CRC tissue (7.5 kPa; min = 1.1 kPa, max = 68 kPa). Increasing stiffness correlates with the pathological T, N, and M stages of cancer as well as with survival. T1, T2, T3, and T4 had respective median EM values of 2.8, 3.5, 8.8, and 13.8 kPa. In addition, the median EM of patients without distant metastases was 7 kPa, whereas it was 13.6 kPa in patients with metastasis (Table 1). Patients with stiffer tumors also had a shorter disease-free survival than those with less stiff tumors [181].

CD44, ALDH1, ALCAM, and CD133 have been identified as CRC stem cell markers. CD133^+^ colon cancer cells are highly tumorigenic, self-renewing, and capable of tumor formation, whereas CD133^−^ cells are unable to do so [182–184]. Overexpression of Collagen 1 promotes expression of CD133 and BMI1 stem cell markers in CRC [185], and high stiffness enhances the expression of CCSCs markers and is critical for the maintenance of the CSC phenotype [186].

HCT-8 colon cancer cells were cultured on PA gels with different stiffnesses (1, 21, 47 kPa) and on a polystyrene surface with a stiffness of 3.6 GPa. At 21 kPa, a higher proportion of metastatic-like R-cells (rounded, separated, metastatic -like phenotype; more aggressive) was observed in comparison to E cells (cells with an epithelial-like phenotype) [187]. In addition to confirming the E-R transition of HCT-8 cells on a PA scaffold with 20 kPa [174], R cells exhibited higher ALDH3A1 activity as a CSC marker for colon carcinoma and other cancer tissues [188, 189]. Upregulation of TNS4, CLDN2, and AKR1B10 in cells cultured on scaffold with 20 kPa; all of these molecules play key roles in cancer cell migration, invasion, proliferation, and apoptosis [174]. The optimum stiffness for HCT-116 tumor stem cell proliferation and marker expression in PEGDA gels was determined to be 25 kPa (2–70 kPa). HCT-116 cells grown on PEGDA gels (2–70 kPa) showed the highest level of tumor stem cell proliferation and marker expression at 25 kPa [109]. HCT-116 cells were grown on matrices with different stiffness (2–20 kPa); at high matrix stiffness, YAP activation in CRC stem cells was considerably elevated, leading to an increase in stemness marker expression (CD133, ALDH1, and Lgr5). Also, due to collagen deposition, CD133 expression was higher in the ITF of CRC tissue samples. Matrix stiffness regulates and maintains CCSC characteristics via the integrin 1/FAK/YAP pathway [186]. CCD18 cells (colon fibroblast cells) were grown on fibronectin-functionalized PA substrates of 2, 10, 40, 95, and 120 kPa. Concentration of Activin A [190], the molecule that regulate self-renewal, plasticity, differentiation and metastatic potential of CSCs [191], was elevated and reached a plateau at 40 kPa in the conditioned medium of CCD18 cells. CRC epithelial FET cells were treated with conditioned medium from CCD18 cultured on increasing stiffness substrates in order to investigate the functional effects of activin A. The highest migration in cancer cells was detected using conditioned medium of the 40 kPa substrate. CRC cells migrated less after the addition of follistatin, a ligand trap for activin A [190] (Table 2). The elevated Activin A level enhanced invasive ALDH^hi^ CSC-like phenotypes and cancer cell plasticity and metastatic potential [192, 193]. As such, studies have shown that the stiffness of the ECM modulates the frequency and characteristics of CCSCs.

## Clinical implications

Considering the significant role of CSCs in the progression of cancer, therapeutic approaches that fail to eliminate CSCs are likely ineffective [194]. Therefore, it is important to develop novel anticancer strategies that directly target CSC populations [195, 196] or components of the TME that cause CSCs to proliferate, make them more aggressive, and sustain their population. Over the course of previous years, there is a significant body of knowledge addressing the mechanisms and players of TME involved in the maintenance of CSCs. Based on the findings reported in the preceding sections, we conclude that matrix stiffness, a mechanical characteristic of TME, plays a key role in CSCs function. Inhibition of stiffness as a supportive niche for CSCs appears to be one of the most effective cancer treatments and provides a novel therapeutic approach to enhance patient outcomes.

Also, cancer stem cell numbers and functional populations are distinct concepts; in fact, stem cell identity and functionality are different [197, 198]. For instance, each homeostatic mouse colonic crypt includes 5 to 7 functional stem cells [198]. However, the number of cells that express stem cell markers such as Lgr5 is ~ 16 per crypt [199]. The position of CSCs in the TME affects its functionality [200]. Functional CSCs that drive tumor progression mainly reside at the tumor's edge, close to CAFs [201]. Also, functional CSC are not necessarily the same cells that express known-CSC markers. In addition, the TME defines the enrichment of functional CSC cells in response to chemotherapy [201]. Therefore, the TME is dominant over cell-autonomous features in defining stem cell functionality. Hence, cancer therapies could be improved by strategies that particularly target the TME compartment, including its stiffness. This approach can block access to the activating signals that provide the soil for differentiated cells to become clonogens and restrict CSCs from entering their favorable niche.

Several approaches for targeting ECM stiffness in cancer have been explored. Targeted therapies against the factors that contribute to establishment of stiffness or against the stiffness-induced activated signaling pathways could potentially modulate and control the effects of stiffness on CSCs. In the mice treated with collagozome, a liposome encapsulating collagenase, malignant tumors were reduced in size by 87% [202]. Collagenases degrade collagen, allowing for improved drug delivery to tumor sites [203]. Inhibition of LOX activity, one of the well-known molecules for matrix stiffness, reduced tumor progression and metastasis in mice [204]. The drug losartan efficiently suppresses lung tumor metastasis by decreasing the level of LOX [205], and inhibiting collagen I synthesis and deposition (NCT01821729 and NCT04106856). In a model of collagen-dependent lung cancer metastasis, trihydroxyphenolics blocked collagen deposition by inhibiting LOXL2 [206].

After resection of the primary tumor, stiffness-affecting components may cause recurrence and distant metastases [207, 208]. Peritoneal surgery in mice creates hypoxic areas at the surgical site and increases LOX expression, which enters the circulation and lungs. The presence and activity of LOX as well as the expression of fibrillar collagen were considerably elevated in the lungs of surgically treated mice, which led to tumor cell seeding and lung metastasis. Notably, LOX inhibition following surgery reduces metastasis and improves survival [116].

Patients with metastatic CRC are widely treated with anti-VEGF in combination with chemotherapy, but the survival benefit is modest due to acquired resistance [209]. Anti-VEGF therapy, such as bevacizumab, enhanced hyaluronic acid (HA) deposition and stiffness of metastatic liver. The remodeling of the ECM and increased stiffness appears to be driven by treatment-induced hypoxia in the tumor. Stiff ECM decreases blood perfusion, which is a key factor in determining the treatment outcome. In preclinical models, they showed that enzymatic depletion of HA, partially restored perfusion in the liver in metastatic colorectal cancer following chemotherapy and anti-VEGF therapy leading to prolonged survival [210]. These results suggest that factors causing stiffness such as HA could be a potential therapeutic target for reducing physical barriers to systemic treatments in cancer patients receiving anti-VEGF therapy.

Volociximab is a monoclonal antibody used to target integrin α5β1 and reduce ECM stiffness in several tumors [211]. Cilengitide, as an α6β5 integrin inhibitor, reduced the progression of malignancies in a variety of preclinical studies, leading to its investigation in clinical trials [212]. Focal adhesion kinase (FAK) inhibitor defactinib suppressed tumor growth and metastatic ability and increased the overall survival of xenografted animals [213]. YAP plays a significant role in the formation and maintenance of CSCs characteristics as well as the promotion of tumorigenesis, metastasis, and recurrence, several attempts have been conducted to investigate its therapeutic potential [179, 214, 215]. The pro-oncogenic property of the YAP pathway, requires YAP/TEAD binding to activate YAP-dependent downstream signaling [38]. The molecules that impair this binding could be potential therapeutic agents. Verteporfin is an inhibitor of YAP/TEAD interaction that suppressed the CSC-associated characteristics of gastric cancer cell line and inhibited tumor growth in a xenograft model [216]. In addition, VGLL4 competes with YAP for binding with TEAD, which suppresses cancer [217]. These results showed the therapeutic potential of YAP for modulating CSC characteristics. Also, the activity of TAZ, another key mechanotransducer, can affect CSCs [218, 219]. Mechanical cues can lead to the formation of a transcriptional complex of TAZ and TEAD4 and the expression of SOX2, which modulates the maintenance and self-renewal of CSCs [218]. These findings suggest that targeting the TAZ-SOX2 axis could be a potential treatment for cancer. The activation of TAZ is required for breast CSCs to maintain their self-renewal and tumor-initiation capacities [52]. In addition, TAZ activation could drive non-CSCs into cells with tumor initiating and self-renewal potential [52], and loss of TAZ impairs the invasiveness, self-renewal, and tumorigenic capacity [220]. YAP/TAZ promotes autophagy through modulating TBC1D2 gene transcription. Autophagy is crucial for the maintenance of oncogenic characteristics and the acquisition of CSC properties, as well as the promotion of cell plasticity and self-renewal of somatic stem cells via YAP/TAZ [214]. Thus, direct or indirect targeting of YAP/TAZ mechanotransduction may block autophagy and, as a result, reducing CSC populations and rendering these cells less aggressive (Table 3). Furthermore, as a result of enhanced exosome secretion in stiff ECM, stiffness-mediated secreted exosomes and their contents could be potential therapeutic agents [80]. Collectively, in order to increase cancer patients’ survival, it is suggested that novel therapeutic agents, such as those that target stiffness, be used in combination with standard cancer treatments, such as chemotherapy and immunotherapy. Future clinical trials should focus on novel therapeutic agents that target the stiffness of the CSC niche or critical molecules that activate signaling pathways mediated by changes in stiffness.

**Table 3:** Anti-stiffness treatment strategies and results

Target	Pharmacological Agent	Result	Ref.
Collagen	Collagozome	87% reduction in the size of malignant tumors. Enhanced drug delivery to cancer sites	[202, 203]
LOX	Losartan	Reduced tumor progression and metastasis. Suppressed lung tumor metastasis	[204, 205]
α5β1 integrin	Volociximab	In bone metastasis or tumorigenesis models, Volociximab (M200) significantly reduced tumor outgrowth and blunted cancer-associated bone destruction	[211]
α6β5 integrin	Cilengitide	In a variety of pre-clinical studies, the drug reduced the progression of tumors, which led to its study in clinical trials	[212]
FAK	Defactinib	Defactinib suppressed tumor growth and metastasis in xenografted animals and enhanced their overall survival	[213]
YAP/TEAD	VGLL4	VGLL4 suppresses Human Gastric Cancer Tumor Growth	[217]
YAP/TEAD	Verteporfin	Suppressed the CSC-associated characteristics of gastric cancer cell line and inhibited tumor growth in a xenograft model	[216]
YAP	Simvastatin	Simvastatin could inhibit cancer cell proliferation, migration, and invasion and promote apoptosis	[215]

## Future directions

We have progressed in our understanding of the complex molecular mechanisms whereby matrix stiffness influences CSCs, but important questions remain.

### How long does the tumor cell's mechanical memory persist?

Over time, cells store information on past mechanical cues, and this mechanical memory can influence the initiation of metastasis. The transfer of mesenchymal stem cells from a stiff matrix to a soft matrix blocked the re-localization of YAP from the nucleus to the cytoplasm for up to 10 days [221]. This mechanical memory effect suggests that the optimum time to initiate treatment with stiffness-reducing medications must be considered and determined.

### Treatment based on heterogeneity of stiffness

As mentioned above section, some tumors display mechanical heterogeneity, and ITF is stiffer than the tumor's core. This heterogeneity can affect tumor cell activity in various ways. Cell in core have higher proliferation, glycolytic metabolism, whereas cell in peripheral zones have increased MMP9, and OXPHOS and FA metabolism [4]. Also, the distribution of CSCs correlates with this mechanical heterogeneity, such that ITF has the highest proportion of CSCs [5]. As such, the therapeutic strategy could be based on the local maps of ECM stiffness and its function.

### And from a different perspective, may the stiffness of the cancer tissues serve as a platform for CSC-targeted therapy?

**Fig. 5:**
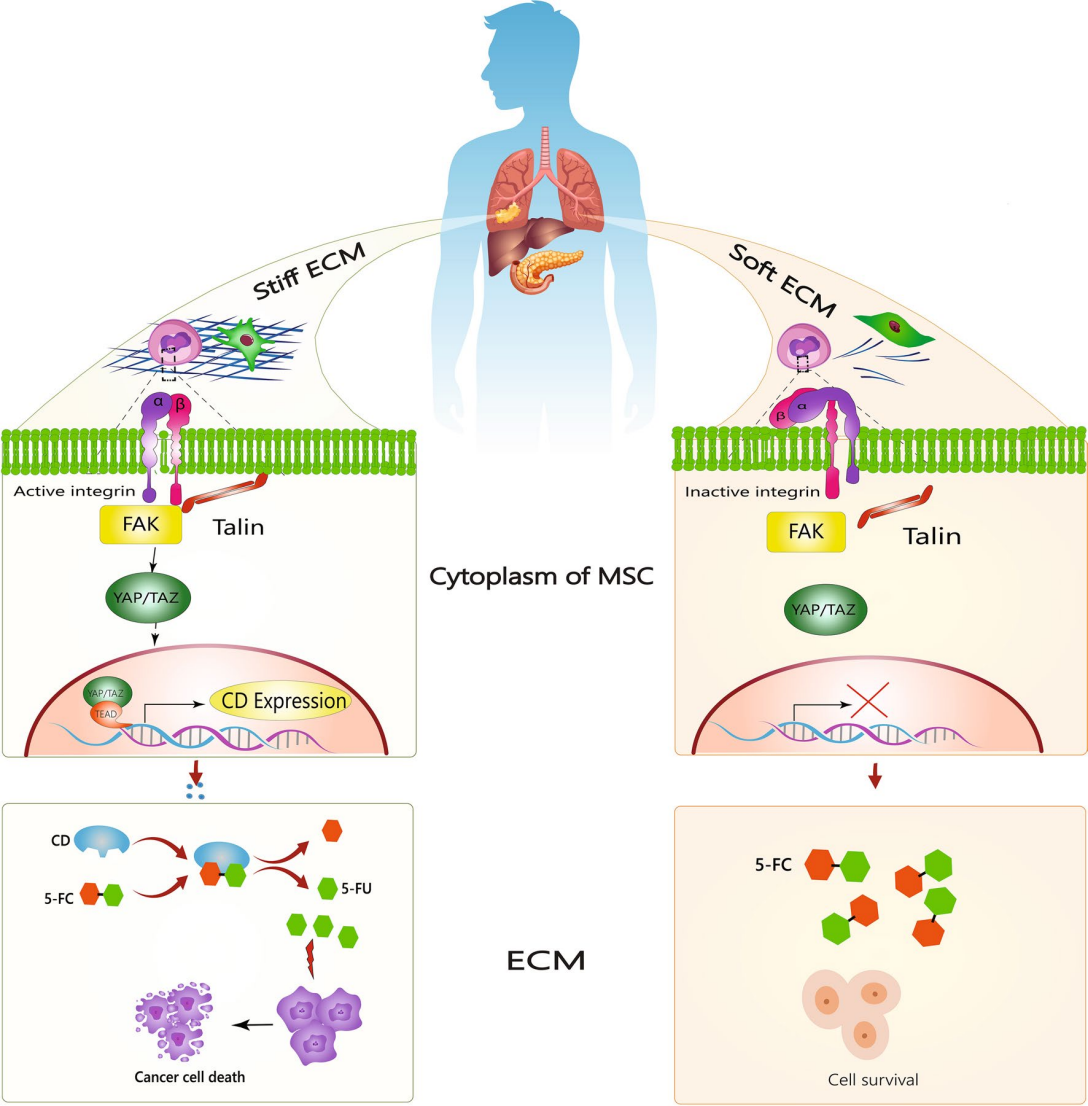
The matrix’s stiffness as a platform for targeted therapy. Mesenchymal stem cells that have a mechanosensitive promoter- driven -based vectors (MSCs) are used to develop a mechanoresponsive cell system (MRCS). In response to increased matrix stiffness, engineered MSCs, selectively home to and target cancer metastases. In stiff ECM, YAP of MRCS localizes to the nucleus, and cytosine deaminase (CD) is expressed. In the tumor microenvironment (TME), CD converts the prodrug 5-fluorocytosine (5-FC) to the active drug 5-fluorouracil (5-FU), which leads to the death of cancer cells. In soft ECM, MSC YAP localizes to the cytoplasm and inhibits CD transcription

The stiffness index can serve as a platform to convert the pro-drug into a drug molecule for the targeted therapy of cancer cells and probably CSCs. Liu et al. designed a mechanoresponsive cell system (MRCS) that uses the specific stiffness index in the TME to target and treat cancer metastases selectively [222]. It is known that infused mesenchymal stem cells (MSC), selectively home to tumors and metastatic sites, in response to increased matrix stiffness [223]. Hence, MSCs can be utilized to designed a MRCS that have a mechanosensitive promoter–driven -based vectors. In stiff matrix, YAP of MRCS localizes to the nucleus, and cytosine deaminase (CD) is expressed, CD converts the prodrug 5-fluorocytosine (5-FC) to the active drug 5-fluorouracil (5-FU) at the metastatic site, which leads to the death of cancer cells [222]. In soft matrix, MSC YAP localizes to the cytoplasm and inhibits CD transcription. This shows that the stiffness of the matrix can serve as a platform for targeted therapies and enables the efficient delivery of drugs to the target site (Fig. 5).

The surgical removal of primary tumors is associated with the formation of scars, which are stiffer than healthy tissue [116]. Is there a possibility that surgery-induced stiffness contributes to the tumor recurrence through inducing stem cell features? Is the stiffness of the matrix formed during tumorigenesis and the stiffness caused by surgery a more suitable niche for CSCs to enter a dormant state? If so, does stiffness affect the period of dormancy for these CSCs?

## Conclusions

A plenty of evidence suggest that the stiffness of tumor matrix is significantly higher than that of normal tissues and strongly correlates with disease progression, metastasis and clinical outcomes in a range of cancers, including BC, CRC, HCC, and PDAC [9, 224]. Matrix stiffness not only plays a role in the transformation of tumor cells into CSCs but can serve as a means of sustainment of the CSC niches, hence promoting and maintaining particular CSC characteristics. Recent progress in understanding the molecular biology of tumor stiffness, particularly its effect on CSC biology, has provided an alternative explanation for tumor development, metastasis, and prospective therapies. Herein, we propose that the importance of tumor matrix stiffness in CSCs can provide insight into novel cancer therapy strategies. All factors that create matrix stiffness, such as LOX, and signaling pathways that are modulated by matrix stiffness, such as YAP, seem to be viable candidates for therapeutic approaches.

## Data Availability

Not applicable.

## Abbreviations

AFM: Atomic force microscopy
ALDH: Aldehyde dehydrogenase
ABCG2: ATP binding cassette subfamily G member 2
ALL: Acute lymphoblastic leukemia
α-SMA: Alpha-smooth muscle actin
AKT: Serine/threonine-protein kinase
BC: Breast cancer
BCSC: Breast CSC
CSC: Cancer stem cell
CAF: Cancer-associated fibroblast
CCC: Cholangiocellular carcinoma
CCSC: Colorectal CSC
CRC: Colorectal cancer
CTC: Circulating tumor cell
DCIS: Ductal carcinoma in situ
ECM: Extracellular matrix
EMT: Epithelial-mesenchymal transition
EpCAM: Epithelial cell adhesion molecule
EM: Elastic modulus
EGFR: Epidermal growth factor receptor
FAK: Focal kinase adhesion
FN1: Fibronectin 1
FA: Fatty acid
GSK3α/β: Glycogen synthase kinase 3α/β
HCC: Hepatocellular carcinoma
HA: Hyaluronic acid
HME: Harmonic motion elastography
ITF: Invasive tumor front
IBC: Invasive breast cancer
IDC: Invasive ductal breast carcinoma
LOX: Lysyl oxidase
LCSC: Liver CSC
MMP: Matrix metalloproteinase
MSC: Mesenchymal stem cell
NSCLC: Non-small-cell lung cancer
OPN: Osteopontin
OXPHOS: Oxidative phosphorylation
PDAC: Pancreatic ductal adenocarcinoma
PI3K: Phosphatidylinositol-3-kinase
PTEN: Phosphatase and tensin homolog
PA: Polyacrylamide
PaCSC: Pancreatic CSC
ROCK: RhoA/Rho-associated protein kinase
TAM: Tumor associated macrophages
TACS: Tumor-associated collagen signatures
TNBC: Triple-negative BC
3D: Three-dimensional
THBS1: Thrombospondin-1
TME: Tumor microenvironment
TAZ: WW domain-containing transcription regulator 1 (WWDR1)
YAP1: Yes-associated protein 1
